# Atopic dermatitis induces the expansion of thymus‐derived regulatory T cells exhibiting a Th2‐like phenotype in mice

**DOI:** 10.1111/jcmm.12806

**Published:** 2016-03-02

**Authors:** Verena Moosbrugger‐Martinz, Christoph H. Tripp, Björn E. Clausen, Matthias Schmuth, Sandrine Dubrac

**Affiliations:** ^1^ Department of Dermatology, Venereology and AllergologyMedical University of InnsbruckInnsbruckAustria; ^2^ Institute for Molecular MedicineUniversity Medical Center of the Johannes Gutenberg‐University MainzMainzGermany

**Keywords:** atopic dermatitis, regulatory T cells, thymic stromal lymphopoietin, vitamin D3

## Abstract

Atopic dermatitis (AD) is a widespread inflammatory skin disease with an early onset, characterized by pruritus, eczematous lesions and skin dryness. This chronic relapsing disease is believed to be primarily a result of a defective epidermal barrier function associated with genetic susceptibility, immune hyper‐responsiveness of the skin and environmental factors. Although the important role of abnormal immune reactivity in the pathogenesis of AD is widely accepted, the role of regulatory T cells (T_regs_) remains elusive. We found that the T_reg_ population is expanded in a mouse model of AD, i.e. mice topically treated with vitamin D3 (VitD). Moreover, mice with AD‐like symptoms exhibit increased inducible T‐cell costimulator (ICOS)‐, cytotoxic T‐lymphocyte antigen‐4 (CTLA‐4)‐ and Glycoprotein‐A repetitions predominant receptor (GARP)‐expressing T_regs_ in skin‐draining lymph nodes. Importantly, the differentiation of T_regs_ into thymus‐derived T_regs_ is favoured in our mouse model of AD. Emigrated skin‐derived dendritic cells are required for T_reg_ induction and Langerhans cells are responsible for the biased expansion of thymus‐derived T_regs_. Intriguingly, thymus‐derived T_regs_ isolated from mice with AD‐like symptoms exhibit a Th2 cytokine profile. Thus, AD might favour the expansion of pathogenic T_regs_ able to produce Th2 cytokines and to promote the disease instead of alleviating symptoms.

## Introduction

Atopic dermatitis (AD) is one of the most common inflammatory skin conditions, predominantly affecting infants and children. It is characterized by pruritus, eczematous lesions and skin dryness. Atopic dermatitis is a complex chronic relapsing inflammatory skin disorder involving immune hyper‐responsiveness of the skin, epidermal barrier abnormalities, genetic susceptibility and environmental factors 1. In its acute phase, AD is characterized by an abnormal production of thymic stromal lymphopoietin (TSLP), an alarmin secreted by keratinocytes and leading to expansion of Th2 cells *via* activation of Langerhans cells (LCs) 2, 3, 4. Moreover, interleukin (IL)‐17 has been shown to be present in acute AD 5. A Th1/Th22 predominant immune response, dermal infiltration with inflammatory dendritic epidermal cells (IDECs), macrophages and eosinophils as well as bacterial superinfection are classical features of the chronic phase of AD 2.

Regulatory T cells (T_regs_) play a critical role in the maintenance of peripheral tolerance and in the control of allergic responses. Despite a growing interest in the role of T_regs_ in the pathogenesis of AD, their precise role remains unclear. In both humans and mice, loss‐of‐function mutations in the FoxP3 gene lead to a multiorgan inflammatory response including skin inflammation resembling AD associated with elevated serum IgE levels, eosinophilia, allergic airway inflammation, food allergies and other autoimmune symptoms 6, 7. These data suggest that lack of functional T_regs_ is sufficient to recapitulate important immunologic features of AD. Accordingly, lower circulating T_regs_ at birth and lower T_reg_ numbers in cord blood predict a higher risk for the development of AD in the first year of life 8. Furthermore, analysis of thymic tissue from atopic children revealed significantly delayed maturation of thymic T_regs_ as compared to age‐matched, non‐atopic controls 9. However, studies in adults seem to dismiss this hypothesis. Some authors reported equal levels of circulating T_regs_ 10, 11, 12, whereas others found increased circulating T_regs_, directly correlating with AD disease severity in patients with persisting AD in adulthood as compared to healthy controls 13, 14, 15, 16. Furthermore, there are conflicting reports about the presence of T_regs_ in inflammatory infiltrates of AD skin 10, 17, 18, and controversy exists about the immune suppressive capacity of T_regs_ in AD. T_regs_ from AD patients were shown to exhibit normal suppressive activity at baseline 11, 15 or after allergen‐stimulation 19, but T_regs_ exhibiting reduced suppressive function have also been identified in patients with AD 13. Intriguingly, stimulation with staphylococcal superantigen (staphylococcal enterotoxin B) leads to a Th2‐dominated cytokine profile in circulating CCR6^−^ T_regs_ of AD patients 12, 14 and there is emerging evidence that T_regs_ can convert to Th2 cells, thereby contributing to AD instead of dampening the immune response 20.

Because the T_reg_ population remains poorly characterized in AD, we here studied the phenotype and the dynamics of thymus‐derived *versus* peripherally derived T_regs_. Dendritic cells (DCs) are professional antigen‐presenting cells and key players in regulating immunity and tolerance, including the instruction of T_regs_. In light of the association of LCs with AD, we also investigated the role of skin‐derived DCs in activating these cells.

## Materials and methods

### Animals

Mice of inbred Balb/c and C57BL/6 strains were purchased from Charles River Laboratories (Sulzfeld, Germany). Mice expressing a diphtheria toxin receptor (DTR) under the control of the Langerin (CD207) gene were bred on a C57BL/6 background as described earlier 21. All mice were used at 2–4 months of age and animal experiments were carried out according to governmental guidelines.

### Mouse treatments

1α,25‐dihydroxyvitamin D_3_ (1 nmol/ear) was dissolved in ethanol. Vehicle (ethanol) or vitamin D3 (VitD) were topically applied once daily onto inner and outer surfaces of mouse ears (10 μl/ear side) over a time period of 10 days (4 days treatment, 3 days no treatment, 3 days treatment) as described earlier 22. Diphtheria toxin in PBS or PBS alone was injected intraperitoneally into Langerin‐DTR mice on day −2 (1 μg/mouse), day +2 (100 ng/mouse), day +6 (100 ng/mouse) and day +8 (100 ng/mouse).

### Antibodies and reagents

Directly labelled primary monoclonal antibodies (mAb) specific for mouse CD4, CD25, inducible T‐cell costimulator (ICOS), CD11c, CCR7, IL‐10 and MHCII were purchased from BD Biosciences (San Diego, CA, USA), and for detection of mouse CCR7, PD‐L1, ICOS L, GITR L, CD11c, Glycoprotein‐A repetitions predominant receptor (GARP) and IL‐13 from eBioscience (San Diego, CA, USA). Directly labelled mAb for detection of cytotoxic T‐lymphocyte antigen (CTLA)‐4 was purchased from Biolegend (San Diego, CA, USA). For intracellular staining with anti‐mouse mAb against FoxP3 (eBioscience) and anti‐mouse mAb against Helios (Biolegend) cells were permeabilized and stained according to the manufacturer's instructions. Cell viability was assessed by LIVE/DEAD Fixable Dead Cell Stain Kit (Invitrogen, Carlsbad, CA, USA) or Fixable Viability Dye (eBioscience). Biotinylated mAb against mouse CD103 and CD25 were purchased from BD‐Biosciences, streptavidin PerCP Cy5.5 from Biolegend, and streptavidin APC from BD Biosciences. Directly labelled mAb against mouse Langerin (clone 929F.3) was purchased from Dendritics (Lyon, France) and used after permeabilization with Cytofix/Cytoperm kit (BD Biosciences), according to the manufacturer's instruction. Purified mAb against IDO was purchased from Biolegend and detected with directly conjugated goat anti‐rat immunoglobulin from BD Biosciences. 1α,25‐dihydroxyvitamin D_3_ and DT were purchased from Sigma‐Aldrich (St Louis, MO, USA).

### Analysis of DCs and lymphocytes in skin‐draining lymph nodes of mice

Auricular skin‐draining lymph nodes (sdLNs) were collected from mice treated with VitD or vehicle on their ears and digested with collagenase D (Roche Diagnostics, Indianapolis, IN, USA) and DNase (Roche Diagnostics) for 25 min. at 37°C. Resulting single cell suspensions were counted in the haemocytometer, stained with mAb and analysed using flow cytometry as previously described 4. Absolute cell numbers per auricular draining lymph node (LN) were calculated on the basis of flow cytometry analysis and haemacytometer cell counts. Mouse T_regs_ were identified by expression of CD4, CD25 and FoxP3, and distinction between induced and natural T_regs_ was made on the basis of Helios staining. CD11c^+^ CCR7^+^ cells in sdLNs were considered as emigrated DCs. Expression of CD103 was used to discriminate epidermal LCs (CD11c^+^ CCR7^+^ CD103^−^ Langerin^+^) from Langerin^+^ dermal DCs (CD11c^+^ CCR7^+^ CD103^+^ Langerin^+^). CD11c^+^ CCR7^+^ CD103^+/−^ Langerin^−^ cells were considered as Langerin^−^ dermal DCs and CD11c^+^ CCR7^−^ CD103^+/−^ Langerin^−^ cells as ‘other DC’.

### Detection of intracellular cytokines

Isolated LN cells were cultured for 4 hrs with 1 μg/ml brefeldin A to block cytokine release; then stained and analysed by flow cytometry.

### Flow cytometry and immunohistochemistry

Flow cytometry analysis was performed on a FACScalibur using CellQuest software (BD Immunocytometry Systems, San Jose, CA, USA) and results were analysed by FlowJo software (Tree Star, Ashland, OR, USA). Five‐ and six‐colour stainings were carried out with a FACScanto using FACSDiva software (BD Immunocytometry Systems). Epidermal sheets were separated from ear skin with 0.5 M ammonium thiocyanate (Merck, Westchester, PA, USA) as described previously 4, washed and stained with antimouse MHC‐class II‐FITC mAb for 1 hr at 37°C. Stainings were visualized by an Olympus BX60 epifluorescence microscope using a 40× objective. Fluorochrome‐ and isotype‐matched immunoglobulins of irrelevant specificity served as negative controls.

### Statistical analysis

Results are shown as mean ± SD, *n* represents the number of mice used per group. Data were analysed using a Student's *t*‐test for normally distributed values or a Mann–Whitney *U*‐test, when values did not show a Gaussian distribution or when *n* < 5.

## Results

### AD‐like inflammation is associated with increased numbers of T_regs_

**Figure 1:**
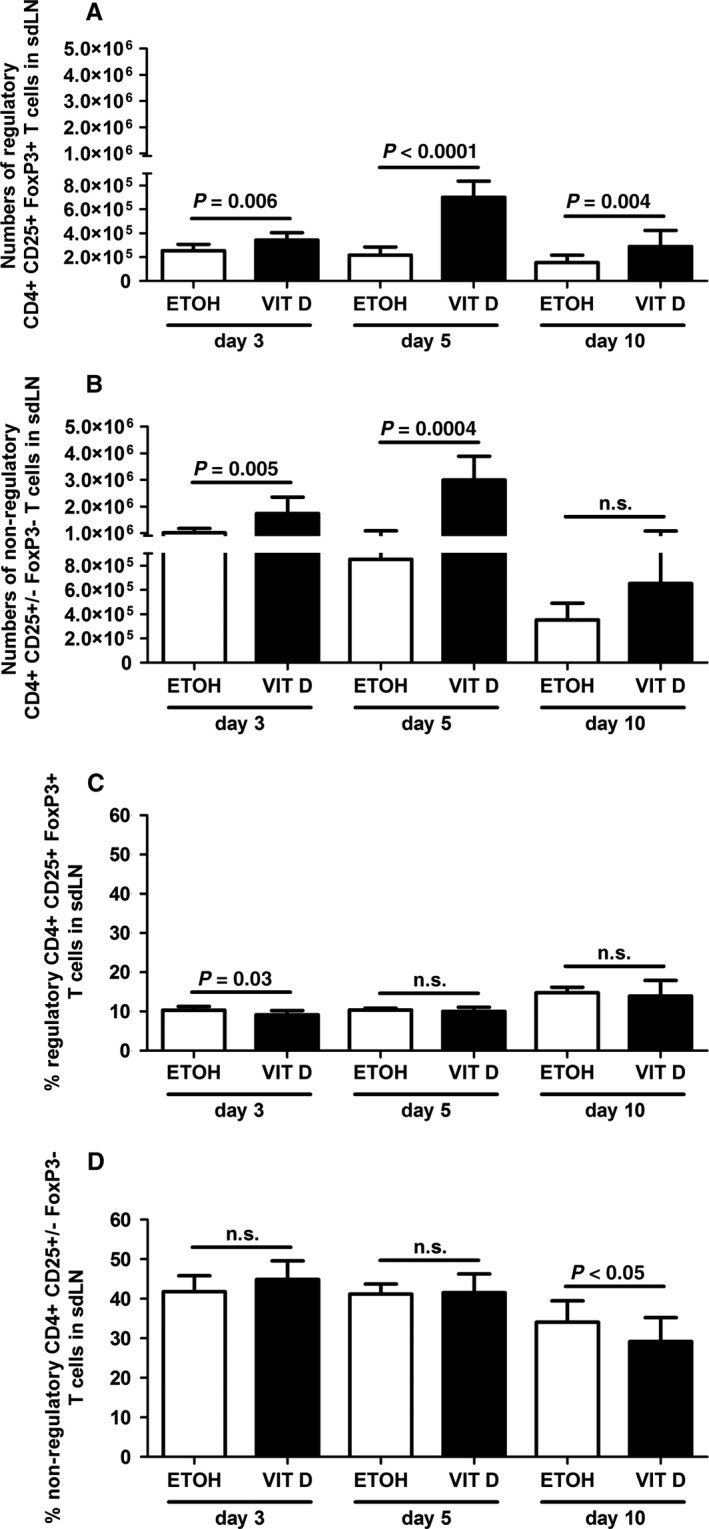
Regulatory T cells are increased in murine AD‐like inflammation. Numbers (**A** and **B**) and percentages (**C** and **D**) of CD4^+^ CD25^+^ FoxP3^+^ T_regs_ and CD4^+^ CD25^+/−^ FoxP3^−^ non‐regulatory T cells in sdLNs of vehicle (ethanol, ETOH) *versus* vitamin D3 (VIT D)‐treated mice on day 3, day 5 and day 10 of treatment. Data are representative of two to three independent experiments and were analysed with a Student's *t*‐test or a Mann–Whitney *U*‐test, *n* = 4–14. n.s. not significant.

To study T_regs_ in AD, we topically treated mice with VitD to trigger high TSLP expression in the epidermis as observed in AD lesions 4, 22. The inflammatory phenotype in these mice is similar to that observed in other TSLP‐overexpressing mice and is characterized by an AD‐like cutaneous inflammation containing Th2 CD4^+^ T cells expressing cutaneous homing receptors and by elevated serum IgE levels 4, 22, 23, 24, 25. Figure 1 depicts the kinetic of T_reg_ (CD4^+^ CD25^+^ FoxP3^+^) and non‐regulatory (CD4^+^ CD25^+/−^ FoxP3^−^) CD4^+^ cell expansion in sdLNs of mice upon treatments. Results show that VitD treatment significantly enhanced numbers of T_regs_ at all time‐points when compared to vehicle treatment (Fig. 1A). In contrast, numbers of other CD4^+^ lymphocytes increased on days 3 and 5 but not on day 10 in sdLNs of VitD‐treated mice when compared to controls (Fig. 1B). Maximal cell numbers were reached for both subsets on day 5 (Fig. 1A and B). Thus, although the expansion of T_regs_ was continuously promoted by VitD, the expansion of other CD4^+^ lymphocytes regressed between day 5 and day 10 (Fig. 1A and B). The variations in cell percentages (Fig. 1C and D) with decreased percentages of T_regs_ on day 3 and decreased percentages of other CD4^+^ lymphocytes on day 10 additionally suggest that the expansion of non‐regulatory CD4^+^ lymphocytes precedes the expansion of T_regs_. Because AD‐like symptoms in mice treated with VitD enhance over time, our data show that expansion of T_regs_ parallels symptom development in this AD model.

### T_regs_ display an activated phenotype in AD‐like inflammation

**Figure 2:**
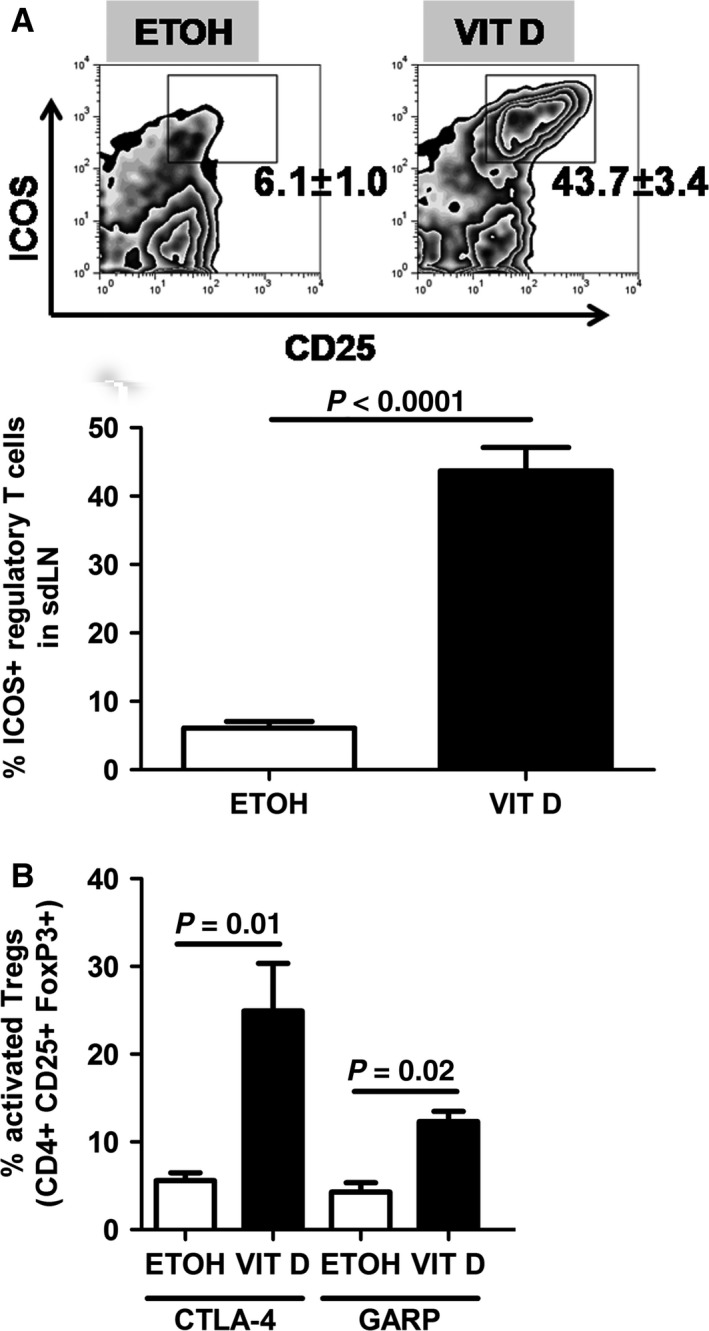
Regulatory T cells are activated in murine AD‐like inflammation. Expression of ICOS (**A**), CTLA‐4 and GARP (**B**) at the cell surface of CD4^+^ FoxP3^+^ T_regs_ in sdLNs of ETOH *versus* VIT D‐treated mice on day 10 of treatment. Data are representative of one independent experiment and were analysed with a Student's *t*‐test or a Mann–Whitney *U*‐test, *n* = 4–6.

To better characterize the CD4^+^ CD25^+^ FoxP3^+^ T_reg_ population in the VitD AD model, we measured the expression of various surface markers which are involved in T_reg_ function in mice with overt AD symptoms, i.e. on day 10 of treatment. Percentages of CD4^+^ CD25^+^ FoxP3^+^ T_regs_ expressing ICOS, CTLA‐4 and GARP at their cell surface were increased in sdLNs of mice with AD when compared to controls (Fig. 2A and B). Hence, peripheral activated T_regs_ are observed in AD‐like inflammation.

### LCs are the first DC subset to emigrate to sdLNs to potentially expand T_regs_ in AD

Earlier work has demonstrated that epidermal TSLP is overexpressed after topical treatment with the VitD analogue MC903 for 4 days 4. As opposed to dermal DCs, LCs acquire an activated phenotype in the skin after MC903 treatment and increased migration to sdLNs 4. These findings suggested that the activation of LCs may be associated with a biased Th2 response prior to the development of clinical signs of AD 4. On the other hand, activation and migration of LCs might also contribute to increased T_reg_ expansion in early AD. We therefore analysed total numbers of CD4^+^ CD25^+^ FoxP3^+^ T_regs_, LCs, Langerin^+^ and Langerin^−^ dermal DCs in sdLNs on days 0, 3 and 5 of topical VitD treatment. Numbers of CD4^+^ CD25^+^ FoxP3^+^ T_regs_ started to significantly increase in sdLNs as early as on day 3 (Fig. S1A), similar to emigrated LCs (Fig. S1B). Numbers of Langerin^+^ and Langerin^−^ dermal DCs and other DCs were not altered on day 3, but started to increase on day 5 (Fig. S1C–E), according to the literature 4, 26, 27. Taken together, our results establish that, in the VitD AD model, LCs are the first skin DC subset to reach the sdLNs, which coincides with the beginning of T_reg_ expansion. It was previously reported that VitD directly induces T_regs_ *in vitro* 28, challenging the requirement of skin‐derived DCs in the development of T_regs_ after topical application of VitD. To address this issue, we removed the application sites (ears) 4 hrs after a single topical application of vehicle or VitD (3 nmol/ear) to prevent any skin DC migration 29. As depicted in Figure S1, removal of the application sites prevented the increase of both T_regs_ (Fig. S1F) and DCs (Fig. S1G) in sdLNs of VitD‐treated as compared to vehicle‐treated mice. We verified that one‐time application of 3 nmol VitD per ear elicits the same effects on the numbers of various DC subsets and T_regs_ in sdLNs than a daily treatment with 1 nmol VitD for 3 days (data not shown). Therefore, expansion of CD4^+^ CD25^+^ FoxP3^+^ T_regs_ is not because of a direct effect of VitD in this mouse model of early AD, but instead requires the migration of skin‐derived DCs to sdLNs.

To further assess the tolerogenic function of skin DCs, we topically applied VitD to mice deficient for Langerin^+^ DCs, including LCs (Fig. S2). Numbers of CD4^+^ CD25^+^ FoxP3^+^ T_regs_ in sdLNs were increased on days 5 and 10 in VitD‐treated mice depleted of Langerin^+^ DCs as compared to vehicle‐treated controls (Fig. S3A and B). Therefore, the overall induction of T_regs_ might be initially dependent on LCs reaching firstly the sdLNs, whereas later on, other skin‐derived DC subsets contribute to this expansion.

### Thymus‐derived T_regs_ are enhanced in AD

**Figure 3:**
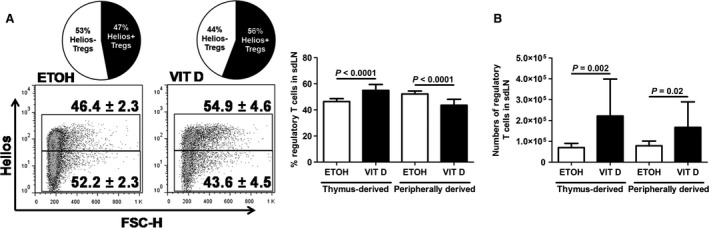
Thymus‐derived regulatory T cells are increased in murine AD‐like inflammation. Percentages (**A**) and numbers (**B**) of thymus‐derived (Helios^+^) and peripherally derived (Helios^−^) regulatory T cells (CD4^+^ (CD25^+^) FoxP3^+^) in sdLNs of ETOH *versus* VIT D‐treated mice on day 10 of treatment. Data are representative of two independent experiments and were analysed with a Student's *t*‐test or a Mann–Whitney *U*‐test, *n* = 10–12.

**Figure 4:**
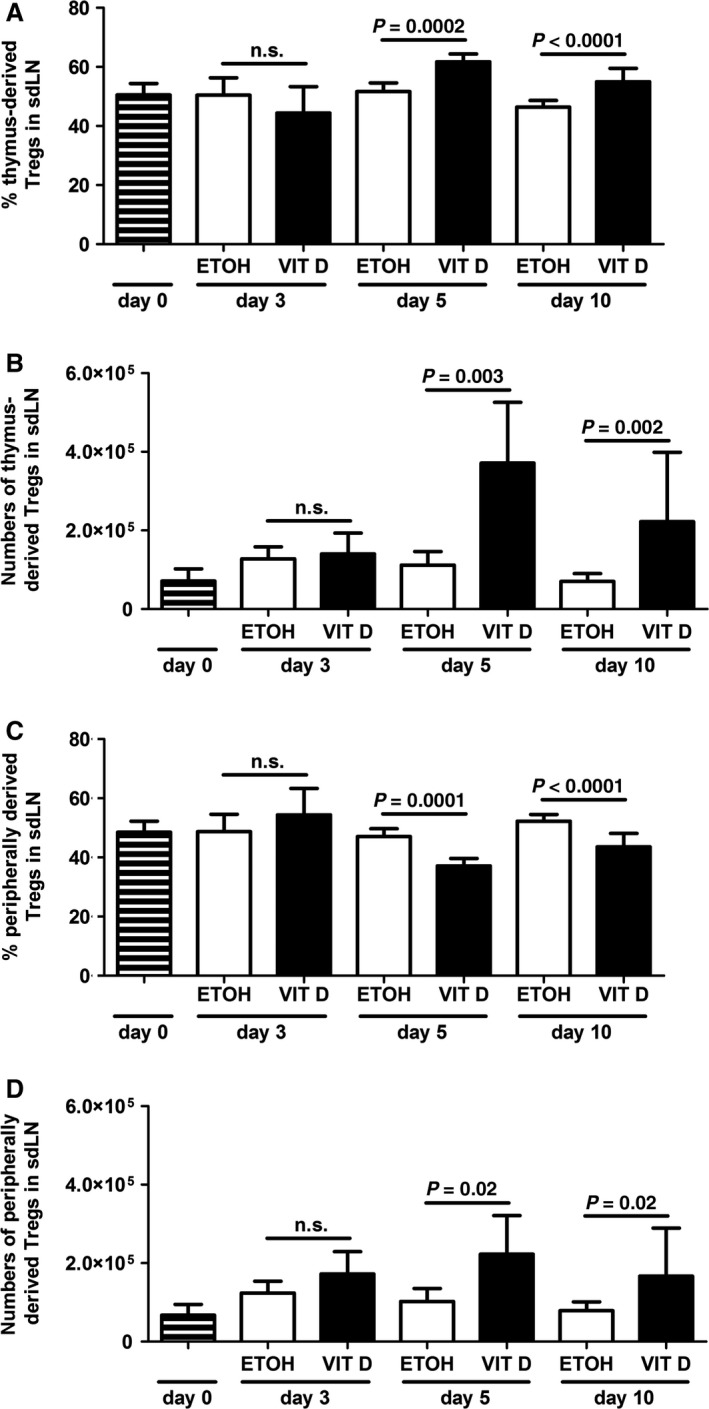
Thymus‐derived regulatory T cells expand in murine AD‐like inflammation. Percentages (**A** and **C**) and numbers (**B** and **D**) of thymus‐derived (Helios^+^) and peripherally derived (Helios^−^) regulatory T cells (CD4^+^ (CD25^+^) FoxP3^+^) in sdLNs of ETOH *versus* VIT D‐treated mice on day 0, day 3, day 5 and day 10 of treatment. Data are representative of two to three independent experiments and were analysed with a Student's *t*‐test or a Mann–Whitney *U*‐test, *n* = 3–12. n.s. not significant.

**Figure 5:**
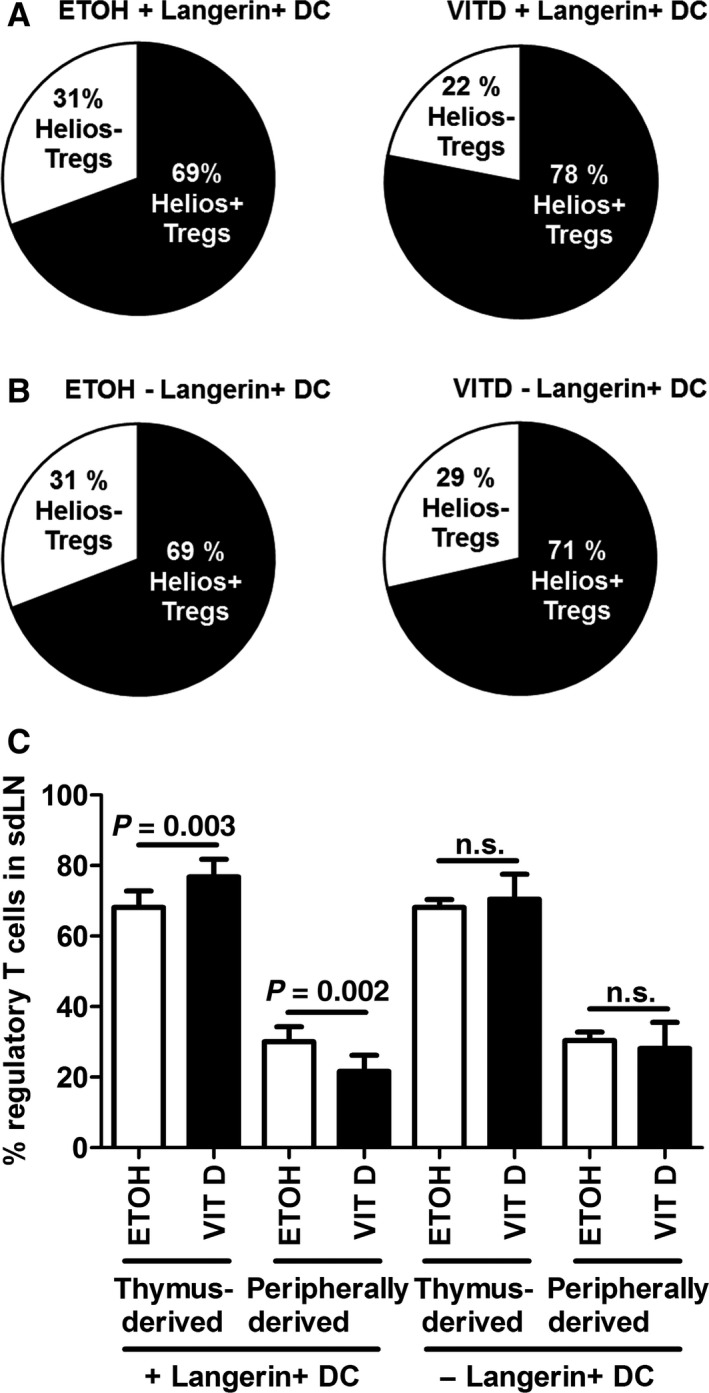
Langerin^+^ dendritic cells expand thymus‐derived regulatory T cells in murine AD‐like inflammation. Percentages of thymus‐derived (Helios^+^) and peripherally derived (Helios^−^) (CD4^+^ CD25^+^ FoxP3^+^) T_regs_ in sdLNs from Langerin‐DTR mice, injected with PBS (+ Langerin^+^ DC) (**A**) or diphtheria toxin (− Langerin^+^ DC) (**B**), at day 5 of treatment. (**C**) Histograms showing all results. Data are representative of three independent experiments and were analysed with a Student's *t*‐test, *n* = 7–8. n.s. not significant.

Helios, an Ikaros transcription factor preferentially expressed in human and mouse CD4^+^ FoxP3^+^ T_regs_, was shown to allow discrimination between thymus‐derived and peripherally induced T_regs_ 30, 31. In VitD‐induced AD‐like inflammation, the percentages of thymus‐derived (Helios^+^) T_regs_ were enhanced, whereas peripheral (Helios^−^) T_regs_ were reduced in sdLNs on day 10 of topical VitD treatment (Fig. 3A). Accordingly, thymus‐derived T_regs_ displayed higher absolute numbers (Fig. 3B). Kinetic analysis revealed a predominant expansion of thymus‐derived T_regs_ in sdLNs of mice as early as day 5 of topical VitD treatment (Fig. 4). Thus, our results indicate an early imbalance of the T_reg_ compartment towards predominating thymus‐derived T_regs_ in AD. Intriguingly, percentages of thymus‐derived T_regs_ failed to increase after depletion of Langerin^+^ DCs in mouse skin at day 5 of VitD‐treatment (Fig. 5). To identify the molecules providing the tolerogenic function to DCs, we screened for expression of various costimulatory and co‐inhibitory molecules, without identifying significant changes in the expression of PD‐L1, ICOS‐L, GITR‐L and IDO by VitD‐exposed DCs (data not shown). Moreover, we detected only trace amounts of IL‐10 production by skin‐derived DCs in our experiments (data not shown). Thus, thymus‐derived T_regs_ are increased in the VitD AD mouse model and Langerin^+^ DCs are required for the early expansion of thymus‐derived T_regs_ in AD, *via* a still elusive mechanism.

### Thymus‐derived T_regs_ exhibit a Th2‐like phenotype in AD

**Figure 6:**
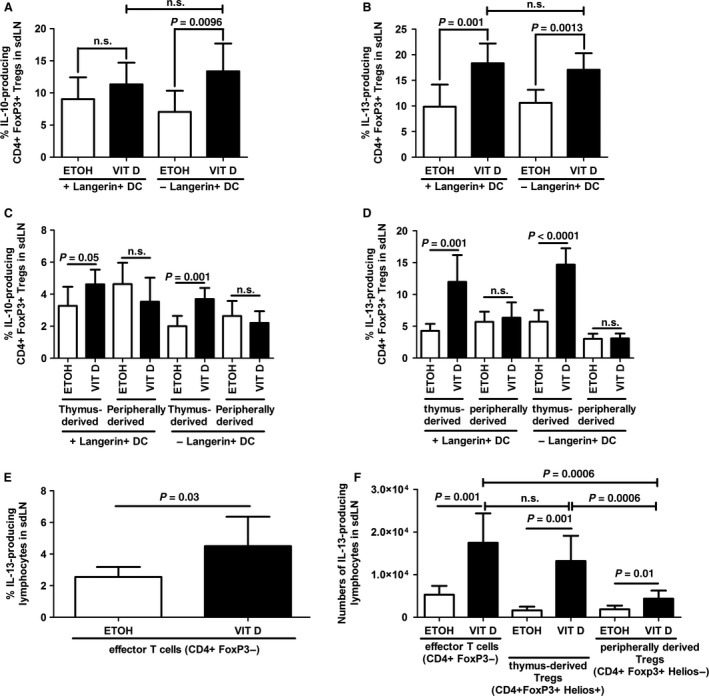
Langerin^+^ dendritic cells are dispensable for cytokine‐producing phenotype of thymus‐derived regulatory T cells in murine AD‐like inflammation. Production of IL‐10 (**A**) and IL‐13 (**B**) by overall CD4^+^ FoxP3^+^ T_regs_ and production of IL‐10 (**C**) and IL‐13 (**D**) by thymus‐ and peripherally derived (CD4^+^ FoxP3^+^) T_regs_ in sdLNs from ETOH *versus* VIT D‐treated Langerin‐DTR mice, injected with PBS (+ Langerin^+^ DC) or diphtheria toxin (− Langerin^+^ DC), at day 5 of treatment. Percentages of effector T cells (**E**) and numbers of effector T cells, thymus‐ and peripherally derived T_regs_ (**F**) producing IL‐13 in sdLNs of ETOH or VIT D‐treated mice, at day 5 of treatment. Data are representative of one to three independent experiments and were analysed with a Student's *t*‐test, *n* = 6–8. n.s. not significant.

We first measured the percentages of overall T_regs_ producing IL‐10 and IL‐13 in the VitD model of AD. T_regs_ isolated from AD mice (day 10 of treatment) produced larger amounts of both IL‐10 and IL‐13 than T_regs_ isolated from healthy controls, regardless of the presence of Langerin^+^ DCs (Fig. S3C and D). In contrast, the production of IL‐13 but not of IL‐10 by T_regs_ was significantly increased earlier during the development of AD, i.e. 5 days after the start of VitD treatment (Fig. 6A and B). Depletion of Langerin^+^ DCs did not alter the production of IL‐13 (Fig. 6B), but increased the secretion of IL‐10 by T_regs_ at early time‐points (Fig. 6A). Moreover, the production of IL‐13 was more strongly induced in T_regs_ than in CD4^+^ effector T cells in VitD‐induced early and overt AD (Fig. S4). To further dissect the production of cytokines within the T_reg_ compartment, we measured percentages of thymus‐ and peripherally derived T_regs_ producing IL‐10 and IL‐13. Thymus‐derived T_regs_ were identified as the main source of IL‐10 and IL‐13 (Fig. 6C–E). Notably, we observed similar numbers of thymus‐derived T_regs_ and effector T cells producing IL‐13 in the VitD AD model (Fig. 6F). Moreover, both numbers of thymus‐derived T_regs_ and effector T cells producing IL‐13 were higher than numbers of peripherally derived T_regs_. These findings emphasize the potential role of thymus‐derived Th2‐polarized T_regs_ in driving the pathogenic events leading to or sustaining AD and suggest that peripherally derived T_regs_ are small contributors to the overall Th2 cytokine production in VitD AD model (Fig. 6D and F). Furthermore, depletion of Langerin^+^ DCs did not alter the production of cytokines by thymus‐derived T_regs_ in the VitD model of AD (Fig. 6C and D). In conclusion, we identified activated, IL‐10‐producing thymus‐derived T_regs_, concomitantly exhibiting a Th2‐like phenotype in the VitD model of AD.

## Discussion

In this study we discovered higher numbers of overall T_regs_ with a specific expansion of thymus‐derived T_regs_ in mice with AD. Furthermore, our results indicate that AD is associated with the expansion of thymus‐derived T_regs_ exhibiting a Th2 phenotype and that LCs seem to be responsible for this biased T_reg_ differentiation. However, other cells or factors from the microenvironment in sdLNs might also contribute to shaping the unusual cytokine profile of thymus‐derived T_regs_. Irrespectively, our data strongly suggest that Th2‐like T_regs_ actively contribute to the development of AD 32.

Several groups reported increased T_regs_ in the peripheral blood 13, 14, 15, 16 and skin lesions 10 of AD patients, whereas others did not 10, 11, 12, 18. In support of the former, we here report increased numbers of CD4^+^ CD25^+^ FoxP3^+^ T_regs_ in sdLNs of mice at different disease stages of AD development (Fig. 1). The T‐cell‐specific costimulatory molecule ICOS is up‐regulated after cell activation and binding to its ligand (ICOS‐L). This step is essential for T_reg_ survival, proliferation and memory rather than for their activation 33. Indeed, reduced numbers of CD4^+^ FoxP3^+^ T_regs_ have been observed in ICOS knockout mice in the steady‐state and upon immunization 34. CTLA‐4 is a CD28 homologue that is up‐regulated after activation of effector T cells and T_regs_. It is associated with T_reg_ suppressive function, although this remains controversial 35. GARP (or LRRC32), a T_reg_‐specific activation marker, is part of the receptor for latency‐associated peptide/latent transforming growth factor‐β complex 36. In the VitD mouse model of AD, percentages of T_regs_ expressing ICOS (Fig. 2A), CTLA‐4 and GARP (Fig. 2B) and producing IL‐10 (Fig. 6A and C, Fig. S3C) were increased, indicating an activated phenotype. However, expansion of activated T_regs_ in VitD AD‐like inflammation is unable to counteract ongoing AD. A similar situation is highly probable in AD patients 10, 13, 14, 15, 16.

T_regs_ can be divided into two subcategories, namely thymus‐derived and peripherally induced T_regs_. While Helios is expressed in all CD4^+^ CD8^−^ FoxP3^+^ mouse thymocytes 30, neuropilin discriminates thymic T_regs_ from peripheral T_regs_ only in the steady‐state 37. Therefore, Helios is currently the most discriminative marker for thymus‐derived T_regs_ 30. We found that the ratio of thymus‐derived (Helios^+^) T_regs_ over peripherally derived (Helios^−^) T_regs_ was increased in sdLNs during AD‐like inflammation (Fig. 3), with enhanced expansion of thymus‐derived (Helios^+^) T_regs_ starting early in the development of the disease (Fig. 4). Thymus‐derived T_regs_ are involved in self‐tolerance and were shown to be activated by microbes 32. Moreover, they are important for the control of Th1 immune responses 38. Thus, thymic T_regs_ might be less efficient at counteracting Th2‐related diseases such as AD when compared to peripherally induced T_regs_.

The T_reg_ population is heterogeneous. Indeed, T_regs_ can acquire alternative effector or hybrid fates, associated with promotion rather than inhibition of inflammation under certain conditions 32. Accordingly, increased production of IL‐5 and IL‐13 has been described in skin‐homing T_regs_ of AD patients 12, 14. High expression of GATA3, as observed in T_regs_ located at barrier sites such as the skin and gut 39, might enable T_regs_ to produce Th2 cytokines. In our VitD AD model, we found increased percentages of IL‐13‐producing T_regs_ in sdLNs (Fig. 6B and D, Figs S3D and S4), similar to Th2 T_regs_ in the skin of AD patients 12, 14. This might potentially confer a pro‐ rather than an anti‐inflammatory phenotype to T_regs_ in AD.

When we analysed the cytokine production by T_regs_ more thoroughly, we found that thymus‐derived T_regs_ were the main source of IL‐10 and IL‐13 in the VitD AD model (Fig. 6C–E). Therefore, we demonstrate here for the first time that expansion of thymus‐derived T_regs_ exhibiting a Th2‐like phenotype is promoted in AD. In fact, the numbers of thymic T_regs_ secreting IL‐13 were similar to the numbers of effector T cells producing IL‐13 (Fig. 6F) and the percentages of thymus‐derived T_regs_ secreting IL‐13 were significantly increased compared to effector T cells (Fig. 6D and E). This strongly suggests a pathogenic role of thymus‐derived Th2‐polarized T_regs_ in AD. Indeed, these T_regs_ might exert poor immunosuppressive properties despite their capacity to produce IL‐10 and consequently contribute to the development of AD‐like inflammation. It would be of particular interest to test this hypothesis by assessing the overall immunosuppressive capacity of these Th2 T_regs_. Unfortunately, due to the nuclear localization of Helios, the lack of Helios‐EGFP mice, and the missing specificity of neuropilin as a surface marker during inflammation, it is not possible to purify Th2 thymus‐derived T_regs_ for further *in vitro* immunosuppressive assays.

Dendritic cells are antigen‐presenting cells regulating immunity and tolerance, respectively, by priming effector T cells and expanding T_regs_ 40. In the VitD AD model, T_reg_ expansion in sdLNs required the presence of skin‐derived DCs (Fig. S1F and G). The time course of T_reg_ induction revealed that, unexpectedly, LCs are the first cutaneous DC subset to reach sdLNs in our experimental setup (Fig. S1A–E). VitD‐induced production of TSLP by keratinocytes might primarily trigger LCs, whereas dermal DCs migrate to sdLNs more quickly after skin immunization with DNFB 4, 41. Previous results established that LCs promote T_reg_ proliferation upon RANK signalling 42. However, depletion of LCs and Langerin^+^ dermal DCs did not affect the size of the overall CD4^+^ CD25^+^ FoxP3^+^ T_reg_ population in sdLNs of VitD‐induced AD mice (Fig. S3A and B) but specifically abolished the induction of thymus‐derived T_regs_ (Fig. 5). Moreover, depletion of LCs and Langerin^+^ dermal DCs did not affect the production of IL‐13 by T_regs_, regardless of Helios expression (Fig. 6B and D, Fig. S3D). Thymus‐derived T_regs_ are involved in antimicrobial responses 32 and LCs are a privileged DC subset sensing microbe‐derived antigens in AD 43. Thus, the expansion of thymus‐derived T_regs_ might be attributed to LCs, while their cytokine production might rather be determined by other cells or factors within the microenvironment of the sdLNs. Langerhans cell‐derived IL‐10 can promote T_reg_ expansion 44, 45, but in our experiments, IL‐10 was not detectable in skin‐derived DCs following VitD treatment (data not shown). Furthermore, expression of PD‐L1, ICOS L, GITR L and IDO by skin‐derived DCs might only have a supporting role in T_reg_ expansion in the VitD AD mouse model (data not shown). Thus, the question how LCs or other DCs promote the expansion of T_regs_ or otherwise impact on their phenotype remains unanswered.

In summary, our work represents the first study demonstrating a preferential expansion of activated CD4^+^ CD25^+^ FoxP3^+^ thymus‐derived T_regs_ exhibiting a Th2 phenotype in a mouse model of AD. Furthermore, differentiation of thymus‐derived T_regs_ seems to depend on LCs, while their cytokine profile might rather be determined by DC phenotype and their microenvironment. Hence, T_regs_ in AD might contribute to the disease rather than playing their role of immunosuppressive cells and thus might represent potential new therapeutic targets.

## Conflicts of interest

Christoph H. Tripp was employed by the COMET Center ONCOTYROL, which is funded by the Austrian Federal Ministries BMVIT/BMWFJ (*via* FFG) and the Standortagentur Tirol. We further appreciate the participation of the TILAK hospital holding company, who serves as a partner in the Oncotyrol research program.

## Supporting information


**Figure S1** (A–E) Numbers of CD4^+^ CD25^+^ FoxP3^+^ T_regs_ (A) and DC (B–E) in sdLNs in mice treated with ETOH or VIT D.
**Figure S2** Depletion of Langerin‐expressing DCs in epidermis (A–D) and sdLNs (E–H) from Langerin‐DTR mice, topically treated with ETOH (A, B, E and F) or VIT D (C, D, G and H), after intraperitoneal injection of PBS (A, C, E and G) or DT (B, D, F and H) on day −2, day +2, day +6 and day +8.
**Figure S3** (A and B) Numbers of T_regs_ in sdLNs from Langerin‐DTR mice, injected with PBS (+ Langerin^+^ DC) or diphtheria toxin (− Langerin^+^ DC), at day 5 (A) and day 10 (B) of treatment.
**Figure S4** Percentages of IL‐13‐producing effector and total T_regs_ in sdLNs of ETOH or VIT D‐treated mice at day 5 (A) and day 10 (B) of treatment.
