# Supplementary material for: Atopic dermatitis induces the expansion of thymus‐derived regulatory T cells exhibiting a Th2‐like phenotype in mice
Source: J Cell Mol Med. 2016 Mar 2;20(5):930–8. doi: 10.1111/jcmm.12806 (PMC4831369; doi:10.1111/jcmm.12806)
Supplement: Supplementary file 1 — Figure S1 (A–E) Numbers of CD4+ CD25+ FoxP3+ Tregs (A) and DC (B–E) in sdLNs in mice treated with ETOH or VIT D. Figure S2 Depletion of Langerin‐expressing DCs in epidermis (A–D) and sdLNs (E–H) from Langerin‐DTR mice, topically treated with ETOH (A, B, E and F) or VIT D (C, D, G and H), after intraperitoneal injection of PBS (A, C, E and G) or DT (B, D, F and H) on day −2, day +2, day +6 and day +8. Figure S3 (A and B) Numbers of Tregs in sdLNs from Langerin‐DTR mice, injected with PBS (+ Langerin+ DC) or diphtheria toxin (− Langerin+ DC), at day 5 (A) and day 10 (B) of treatment. Figure S4 Percentages of IL‐13‐producing effector and total Tregs in sdLNs of ETOH or VIT D‐treated mice at day 5 (A) and day 10 (B) of treatment. [file JCMM-20-930-s001.doc]

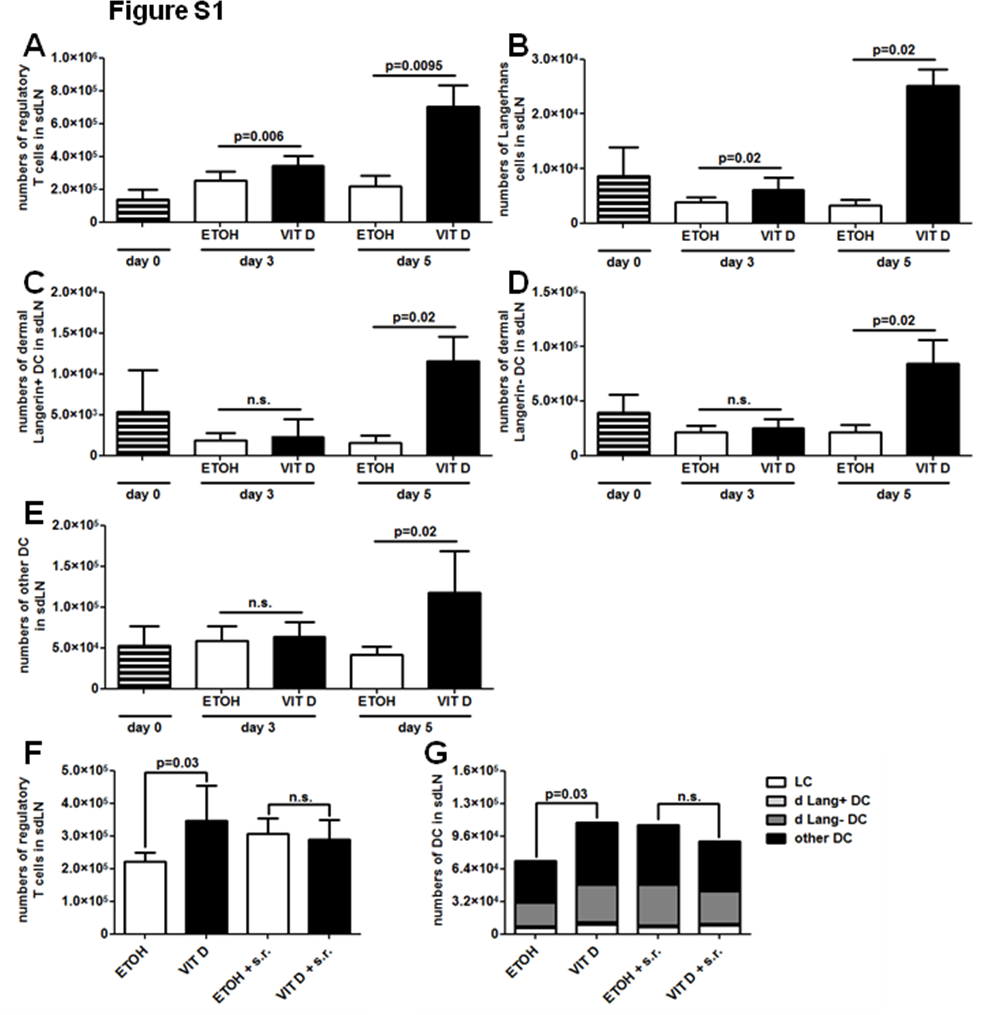


**Fig. S1. (A – E)** Numbers of CD4^+^ CD25^+^ FoxP3^+^ T_regs_ **(A)** and DC **(B – E)** in sdLNs in mice treated with ETOH or VIT D. **(F and G)** Numbers of T_regs_ **(F)** and DCs **(G)** in sdLNs 3 days after removal of the application site (ear) 4h after topical ETOH or VIT D. Data are representative of one to three independent experiments and were analyzed with a Student *t*-test, n=3-10. *LC* Langerhans cells; *dLang+DC* Langerin^+^ dermal DC; *dLang-DC* Langerin^-^ dermal DC; *s.r.*, site removed; *n.s.* not significant.

**
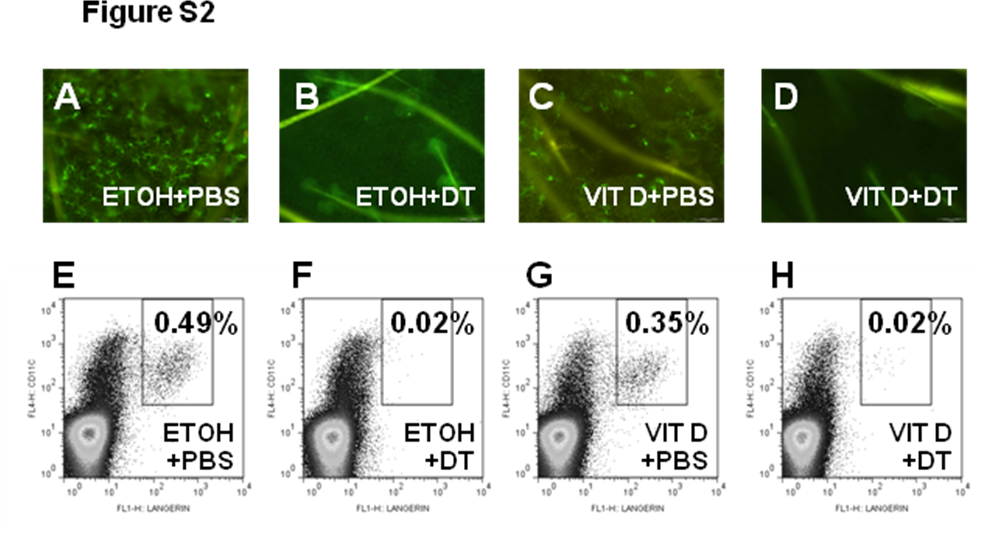
**

**Fig. S2.** Depletion of Langerin-expressing DCs in epidermis **(A**–**D)** and sdLNs **(E**–**H)** from Langerin-DTR mice, topically treated with ETOH **(A**, **B**, **E** and **F)** or VIT D **(C**, **D**, **G** and **H)**, after intraperitoneal injection of PBS **(A**, **C**, **E** and **G)** or DT **(B**, **D**, **F** and **H)** on day -2, day +2, day +6 and day +8.


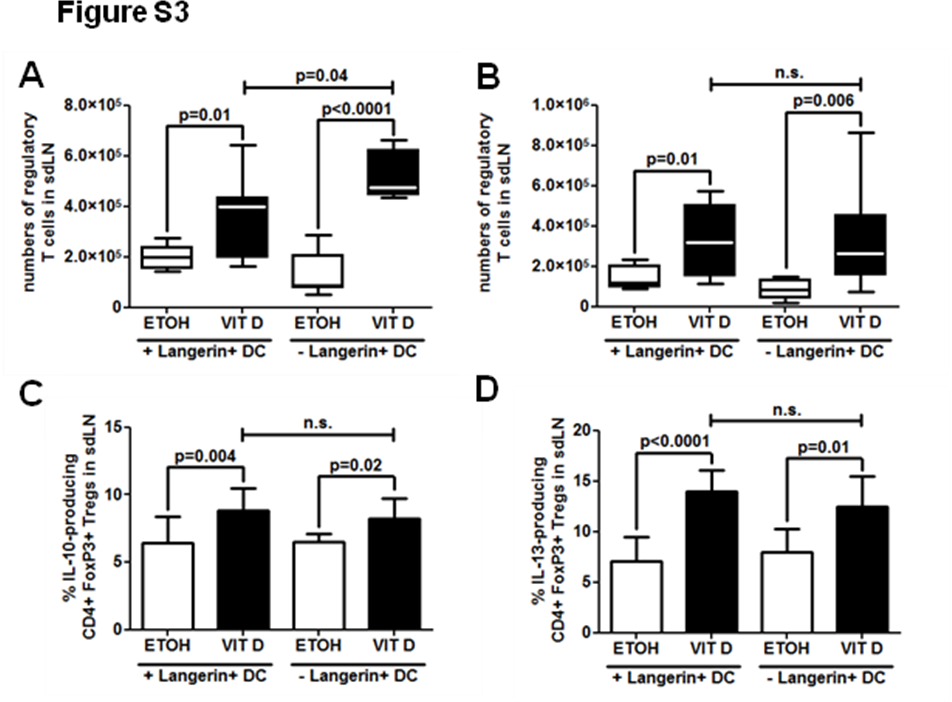


**Fig. S3. (A** and **B)** Numbers of T_regs_ in sdLNs from Langerin-DTR mice, injected with PBS (+ Langerin^+^ DC) or diphtheria toxin (- Langerin^+^ DC), at day 5 **(A)** and day 10 **(B)** of treatment. **(C** and **D)** Percentages of IL-10 **(C)** and IL-13-**(D)** producing CD4^+^ CD25^+^ FoxP3^+^ T_regs_ in sdLNs from Langerin-DTR mice, injected with PBS (+ Langerin+ DC) or diphtheria toxin (- Langerin+ DC), at day 10 of treatment. Data are representative of two independent experiments and were analyzed with a Student *t*-test, n=6-13. *n.s.* not significant.

**
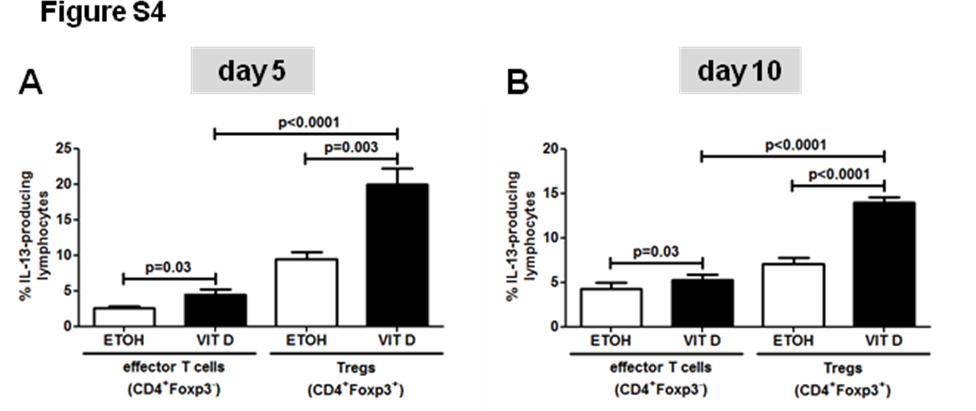
**

**Fig. S4.** Percentages of IL-13-producing effector and total T_regs_ in sdLNs of ETOH or VIT D-treated mice at day 5 **(A)** and day 10 **(B)** of treatment. Data are representative of one to two independent experiments and were analyzed with a Student *t*-test or a Mann-Whitney *U* test, n=6-13.
